# Phosphodiesterase type 5 and cancers: progress and challenges

**DOI:** 10.18632/oncotarget.21837

**Published:** 2017-10-12

**Authors:** Ines Barone, Cinzia Giordano, Daniela Bonofiglio, Sebastiano Andò, Stefania Catalano

**Affiliations:** ^1^ Department of Pharmacy, Health and Nutritional Sciences, University of Calabria, Arcavacata di Rende, Italy; ^2^ Centro Sanitario, University of Calabria, Arcavacata di Rende, CS, Italy

**Keywords:** phosphodiesterase, cancer, targeted therapy, biomarkers, chemoprevention

## Abstract

Cancers are an extraordinarily heterogeneous collection of diseases with distinct genetic profiles and biological features that directly influence response patterns to various treatment strategies as well as clinical outcomes. Nevertheless, our growing understanding of cancer cell biology and tumor progression is gradually leading towards rational, tailored medical treatments designed to destroy cancer cells by exploiting the unique cellular pathways that distinguish them from normal healthy counterparts. Recently, inhibition of the activity of phosphodiesterase type 5 (PDE5) is emerging as a promising approach to restore normal intracellular cyclic guanosine monophosphate (cGMP) signalling, and thereby resulting into the activation of various downstream molecules to inhibit proliferation, motility and invasion of certain cancer cells. In this review, we present an overview of the experimental and clinical evidences highlighting the role of PDE5 in the pathogenesis and prevention of various malignancies. Current data are still not sufficient to draw conclusive statements for cancer patient management, but could provide further rational for testing PDE5-targeting drugs as anticancer agents in clinical settings.

## INTRODUCTION

The current global demographic, epidemiologic and nutritional transitions signal an enormous cancer burden on society in countries of all income levels. The incidence of cancer cases is on continuing growth also because of an increasing prevalence of established risk factors, such as tobacco use, excess body weight, physical inactivity, infection and changes in the reproductive patterns connected with urbanization and economic development. Based on data from GLOBOCAN 2012, an estimated 14.1 million new cancer cases and 8.2 million deaths were recorded in 2012 globally [1]. Breast and lung cancers represent the most frequently diagnosed cancers and the leading causes of cancer death in women and men, respectively, in the world and in less developed countries. Other frequently diagnosed cancers worldwide include those of the prostate, liver, stomach, and colorectum among males and those of the stomach, cervix uteri, and colorectum among females. A substantial proportion of cases can be prevented by broadly adoption of effective measures, such as smoking control, vaccination (for liver and cervical cancers), early detection, and promotion of healthy behaviors. Moreover, the burden of suffering and premature deaths can be decreased through the use of an appropriate therapy and palliative care [2]. Surgery, chemotherapy and radiotherapy are currently the three major treatments to prolong the survival of the majority of cancer patients, but clinical improvements are often associated to undesirable side effects on normal cells or tissues. Thus, as the biology of cancer has become progressively understood on a molecular level, therapeutic research has mainly shifted its focus from cytotoxic oncology drugs to newer target-based agents able to inhibit specifically tumor outgrowth and progression mechanisms or enhance host immune responses against cancer cells. On the other hand, clinical trials and meta-analyses have demonstrated that simultaneous or sequential multi-modal therapies may improve patient outcome, may have acceptable tolerability profiles and may be active against a variety of tumor types as compared with a single-modality therapy [3–6]. In the search of molecularly targeted cancer therapy, tremendous interest has been given to the expression and regulation of the phosphodiesterase type 5 (PDE5) as an important signaling modulator involved in diverse aspects of tumor cell function. In the last decade, a significant number of studies have reported an increased expression of PDE5 in several human cancers compared to normal or surrounding non-neoplastic tissues [7–10]. Concomitantly, PDE5 inhibitors have been examined in multiple malignancies and cancer cell lines for their direct anticancer activities, for their efficacy as chemo-sensitizers and for cancer chemoprevention (reviewed in [11, 12]). In this review, we will highlight the emerging knowledge and our recent findings showing the role of PDE5 as a tumor biomarker as well as a potential target for therapeutic strategies aimed at controlling and eventually curing malignant diseases. First, we will briefly discuss the structure and the biological function of PDE5. Next, we will summarize the significance of PDE5 expression on different cancer types in clinical settings as well as in experimental cellular and animal models. The benefit of targeting PDE5 in cancer prevention or treatment will be also discussed because of the numerous advantages of PDE5 inhibitors.

### The superfamily of PDEs

**Figure 1:**
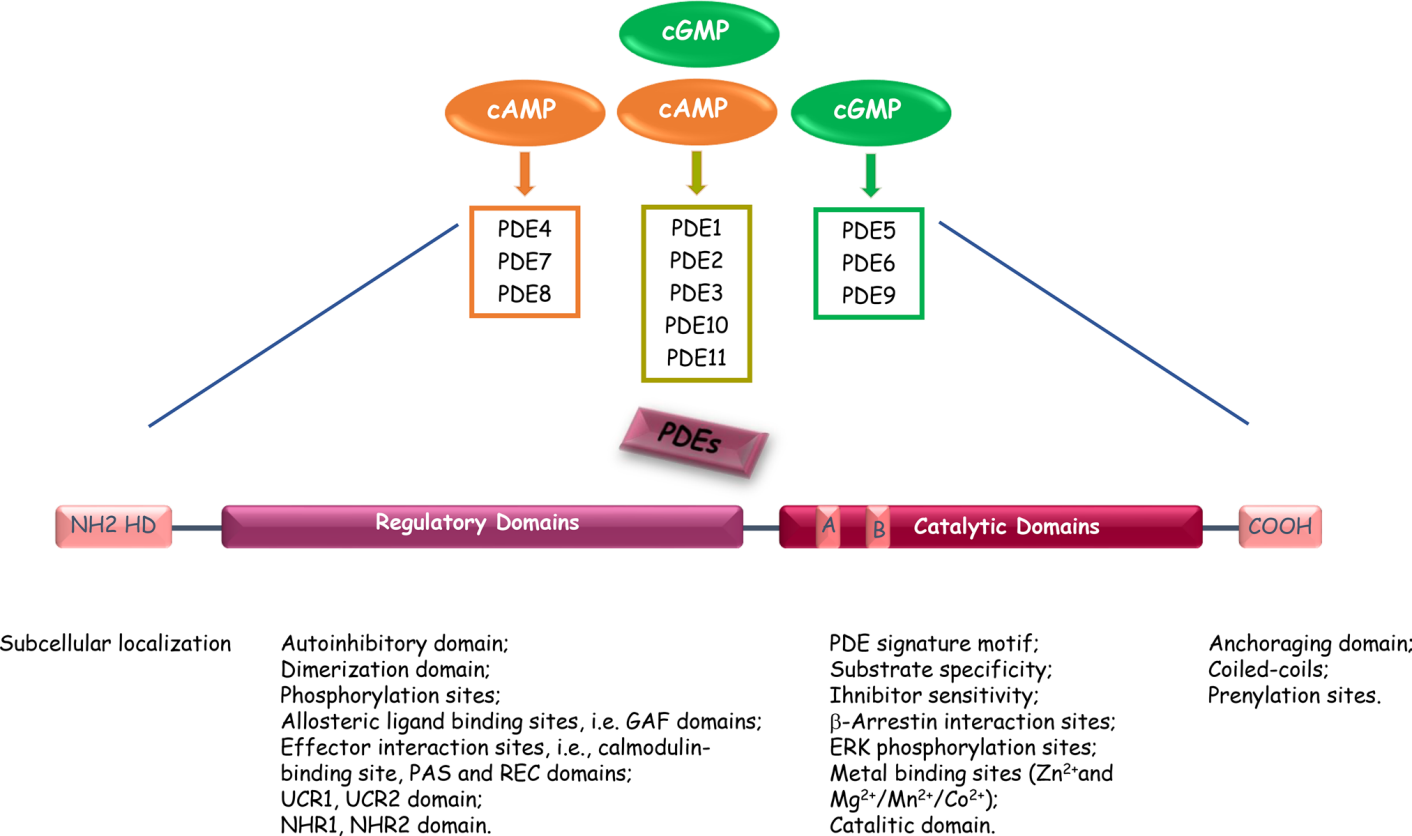
Common structure of the different PDE enzymes The PDE superfamily contains 11 structurally-related gene families. Some PDEs are specific for cAMP or cGMP, and some exhibit dual substrate specificity. The N-terminal portion of the PDEs contains sequences important for cellular localization. The regulatory domain contains PDE-family specific sequences responsible of modulation of PDE enzymatic activities. The catalytic domain is present in the carboxy-terminal part of PDEs and is highly conserved. A and B indicate the two Zn2 + -binding motifs (HX3HXnE/D) that include invariant histidines and are critical to catalysis. HD, Hydrophobic domains.

Mammalian cyclic nucleotide phosphodiesterases (PDEs) constitute a large and complex family of ubiquitously distributed hydrolases that have the unique function of catalysing the hydrolytic breakdown of cyclic adenosine monophosphate (cAMP) and cyclic guanosine monophosphate (cGMP) into the biologically inactive derivates 5′-AMP and 5′-GMP, respectively. The PDE superfamily contains 11 distinct gene families (PDEs 1 to 11), that encode at least 100 distinct PDE isoforms through alternative mRNA splicing, multiple promoters and transcription start sites in human, rat, and mouse [13]. The 11 PDEs can be grouped into three broad categories based on their sequence homology as well as substrate specificity and selectivity. PDE4, PDE7 and PDE8 are specific for cAMP hydrolysis; PDE5, PDE6 and PDE9 are specific for cGMP hydrolysis; PDE1, PDE2, PDE3, PDE10 and PDE11 exhibit dual specificity, acting on cAMP and cGMP with different affinities depending on the isoform (Figure 1 and Table 1) [13–72]. The different PDEs are modular proteins sharing the following common structural organization from N-terminus to C-terminus (Figure 1): 1) a highly divergent regulatory domain in the N-terminal portion; 2) a conserved catalytic core of approximately 270 amino acids (~ 35–50% sequence homology); 3) a region of undetermined function that can be prenylated (PDE6) or phosphorylated (PDE4) in the carboxyl terminus [19]. The N-terminal portions of PDE molecules contain structural determinants and specific amino acid sequences that are responsible for localization of individual PDE isoforms to specific intracellular sites, organelles and membranes as well as for their incorporation into particular multimolecular regulatory complexes or signalosomes. The regulatory regions contain domains that can be subjected to diverse types of modification (e.g., phosphorylation by various protein kinases), or sites that may interact with allosteric ligands (e.g., cGMP binding sites), selective effectors (e.g., Ca^2+^/calmodulin), protein partners (e.g., RAF1), or molecular scaffolds (e.g., caveolin). In addition, N-terminal regulatory regions include dimerization domains and autoinhibitory modules as several PDEs exist as asymmetric homo- or heterodimers (e.g., in PDEs 1, 4, and 5). Informations from the crystal structures of isolated catalytic cores of PDEs have revealed that these domains exhibit the same topography, composed of aminoacids folded into 16 helices [73]. The active site forms a deep hydrophobic pocket that includes a histidine-rich PDE signature sequence motif and consensus metal binding domains, represented by two Zn^2+^ binding motifs (motifs A and B – HX_3_HX_n_E/D) and an additional binding site whose metals could be Mg^2+^, Mn^2+^ or Co^2+^ [73, 74]. In addition to the conserved portions important for cyclic nucleotide and inhibitor bindings, the catalytic domain also encompasses variable determinants that are responsible of PDE family-related substrate affinities and selectiveness [75].

**Table 1:** PDE families and inhibitors


Isozime Family	Gene Members	Substrate Specificity	Regulation	Major Tissue and Cellular Expression	Subcellular Localization	Functions	Inhibitors	References
PDE1	A, B, C	cAMP/ cGMP	Ca2+/ calmodulin	Lung, heart, brain, smooth muscle, testis, sperm, macrophages, lymphocytes	Cytosolic/ perinuclear	Vascular smooth muscle contraction, sperm function (PDE1A); Dopaminergic signaling, immune cell activation, and survival (PDE1B) Vascular smooth muscle cell proliferation, sperm function, neuronal signaling (PDE1C)	Vinpocetine, IC224, IC86.340, SCH51866, 8-MeoM-IBMX, ITI-214	[14–24]
PDE2	A	cAMP/ cGMP	cGMP-stimulated	Adrenal cortex, lung, liver, platelets, heart, brain, macrophages, endothelium	Membrane-bound or cytosolic, mitochondria	Regulates aldosterone secretion, phosphorylation of calcium channel in heart, cGMP in neurons; endothelial cell function under inflammatory conditions	EHNA, BAY60–7550, IC933, PDP, OXIDOLE	[16, 21, 25–29]
PDE3	A, B	cAMP/ cGMP	Phosphorylation/cGMP-inhibited	Lung, heart, adipose tissue, adipocytes, liver, smooth muscle, kidney, hepatocytes, pancreatic beta cells, immune cells, platelets	Membrane-bound or cytosolic	Cardiac contractility, platelet aggregation, vascular smooth muscle contraction, oocyte maturation, renin release (PDE3A) Insulin signaling, cell cycle, proliferation (PDE3B)	Milrinone, Tolafentrine, Enoximone, K-134, Cilostazol, Cilostamide, Trequinsin, OPC-33540	[21, 22, 29, 30–35]
PDE4	A, B, C, D	cAMP	Phosphorylation/cAMP-specfic cGMP-insensitive	Broad, cardiovascular, neural, immune and inflammatory systems	Membrane-bound or cytosolic	Brain function, monocyte and macrophage activation, neutrophil infiltration, vascular smooth muscle proliferation, fertility, vasodilatation, cardiac contractility	Cilomilast, Rolipram, Ro20–1724, Roflumilast, AWD12281, V11294A, SCH35159, GSK256066, Denbufylline, Arofylline, Apremilast	[21, 22, 29, 32, 36–46]
PDE5	A	cGMP	Phosphorylation/cGMP-specific cGMP-activated	Broad, lung, cerebellum, heart, brain, platelets, vascular myocytes, cardiac myocytes, gastrointestinal tissues and penis	Cytosolic	Vascular smooth muscle contraction, platelet aggregation, cGMP signaling in brain	Sildenafil, Taldanafil, DA8159, Exisulind, E402, Vardenafil, Zaprinast, DMPPO, Dipyridamole, Mirodenafil	[16, 21, 29, 47–57]
PDE6	A, B, C, D, G	cGMP	Phosphorylation/cGMP-specific	Retina and pineal gland	Cytosolic	Phototransduction	Avanafil, Udenafil, Zaprinast	[29, 58–61]
PDE7	A, B	cAMP	Transduction activated/ cAMP-specific	Heart, liver, kidney, brain, pancreas, testis, spleen, skeletal muscle, immune cells	Cytosolic	Immune cell activation (PDE7A) Memory function and excreted T (PDE7B)	ASB16165, BRL50481, IC242, Dipyridamole, BMS-586353, Thiadiazoles	[21, 22, 29]
PDE8	A, B	cAMP	cAMP-specfic Rolipram/IBMX insensitive	Broad, testis, liver, heart, kidney, brain, skeletal muscle, thyroid, spleen, colon, ovary, immune cells	Membrane-bound or cytosolic, mitochondria	T-cell activation, sperm or Leydig cell function, T4 and T3 production (PDE8A)	Dipyridamole, PF-04957325	[29, 40, 62–64]
PDE9	A	cGMP	cGMP-specific IBMX/insensitive	Broad, kidney, liver, lung, brain, spleen, prostate, heart	Cytosolic or nuclear	NO-cGMP signaling in brain	BAY 73–6691, SCH-51866, WYQ C28L, PF-04447943	[21, 29, 65, 66]
PDE10	A	cAMP/ cGMP	Unknown	Brain, heart, thyroid, testis	Cytosolic or particulate	Learning and memory	Papaverine, Dipyridamole, PQ-10, TP-10, MP-10	[16, 29, 67, 68]
PDE11	A	cAMP/ cGMP	Unknown	Liver, prostate, testis, salivary and pituitary gland	Cytosolic	Sperm development and function	None selective	[62–72]

As expected from their complex genomic organization, multiple PDE isoforms are expressed in almost all cells (Table 1) [75, 76]. However, some cells are relatively enriched in specific PDEs (e.g., photoreceptor PDE6 exclusively expressed in retina rods and cones and in the pineal gland) as well as some PDE alterations are tightly connected to different pathological conditions (e.g., PDE4B abnormalities have been linked to schizophrenia [77]) [75, 76, 78]. Recently, genetic alterations or overexpression in PDE genes were described to be associated with tumor development. Polymorphisms in the genes encoding PDE8A and PDE11A have been associated with a predisposition to developing certain adrenocortical [79], testicular [69], and prostatic [70] cancers. More importantly, PDE5 overexpression has been reported in several types of cancers [7–10].

### PDE5 structure, regulation, distribution, signalling and function

The human PDE5A gene is located on chromosome 4q264,5 and contains about 23 exons spanning approximately 100 kilobases, with the first three exons being alternative exons encoding the isoform-specific 5’-ends of the PDE5 sequences [80, 81]. Cloning and sequencing of the PDE5A gene showed that these alternative exons are arranged in the order of A1-A3-A2 and are separated by an intron of 434 bp between A1 and A3 and by another intron of 361 bp between A3 and A2 [82, 83]. The human PDE5A gene promoter, located upstream of the three isoform-specific first exons, consists of a 139 bp core with full basal activity, a 308 bp upstream regulatory region, and a 156 bp downstream regulatory region. Each of these two regulatory regions could independently convey the responsiveness of cAMP and cGMP to the core promoter and contained multiple consensus sites for several transcription factors, including Sp1 [83, 84]. The weaker PDE5A2 promoter is 182 bp in length and contains one AP2- and three Sp1-binding sequences [84]. The upstream PDE5A promoter is expected to direct the expression of all three PDE5 isoforms, while the intronic PDE5A2 promoter can only control the expression of the A2 isoform [85].

Human PDE5A1 and A2 transcripts are found to be expressed in almost all tissues, with PDE5A2 being more common, and identifiable in almost all cells cultured from aortic smooth muscle, bladder smooth muscle, urethra smooth muscle, penile smooth muscle, penile endothelium, aortic endothelium, etc. On the contrary, the distribution of human PDE5A3 appears to be restricted to tissues with smooth and/or cardiac muscle component [81]. The three PDE5A isoforms share similar cGMP-catalytic activities and differ only in the N-terminal domain, in which no biochemical or physiological function has been identified. The N-terminal part of PDE5 contains two regulatory GAF domains named as GAF A and B (domains originally found to be present in cGMP-regulated cyclic nucleotide PDEs, certain adenylyl cyclases and the bacterial transcription factor FhlA [86]). The identified functions of these regions are cGMP-mediated allosteric regulation and dimerization of GAF-containing PDEs. Allosteric binding of cGMP facilitates phosphorylation of human PDE5 by protein kinase G (PKG) on serine 102 and this phosphorylation seems to play a role in stabilizing the enzyme in its cGMP-bound active state [87], increasing both its catalytic activity and cGMP-binding affinity [87–90]. In addition of functioning as negative feedback for cGMP signalling by activating the cGMP-specific PDE5, cGMP levels may influence also the activity of non-selective PDE isoenzymes (e.g., PDE2 or PDE3) and thereby modulate the crosstalk between cyclic nucleotide pathways [91]. Modulation of cGMP concentrations is accomplished by cAMP- and cGMP-dependent activation of PDE2 and cGMP-dependent activation of PDE5 [92–94]. cAMP may also increase cGMP levels by inhibiting cGMP-degrading activities of PDE1 and PDE3 [92]. Recently, Zhao et al. demonstrated that amongst all PDEs, PDE2 and PDE5 compensate most strongly for the reduced activity of each other, an event that was indicated as strong coupling [95]. Indeed, NO/cGMP/PKG activity potentiated by PDE5 inhibition is partially compensated by PDE2 and reciprocally, compensatory increase in PDE5 cGMP rates is also the greatest upon PDE2 inhibition. PDE5 inhibition indirectly also leads to a decrease in PDE3 cAMP hydrolysis rates [95]. It was also reported that cGMP is able to directly activate PDE5 without phosphorylation in response to sustained nitric oxide (NO) in the platelet [96]. Moreover, small molecular mass proteins immunologically related to the gamma subunit of PDE6 may prevent PKA-mediated activation of PDE5 in airway smooth muscle [97]. More recently, an elegant study revealed, for the first time in mouse, the existence of three different PDE5A isoforms with similar biochemical features and different distribution patter and highlighted their potential role in the induction of hypertrophy [98]. The authors demonstrated that exogenous overexpression of each variant induced a sustained cell cycle progression in cardiomyocytes and fibroblasts transfected cells, with PDE5A3 isoform being more efficient in the modulation of hypertrophic markers respect to the other mPDE5A isoforms.

**Figure 2:**
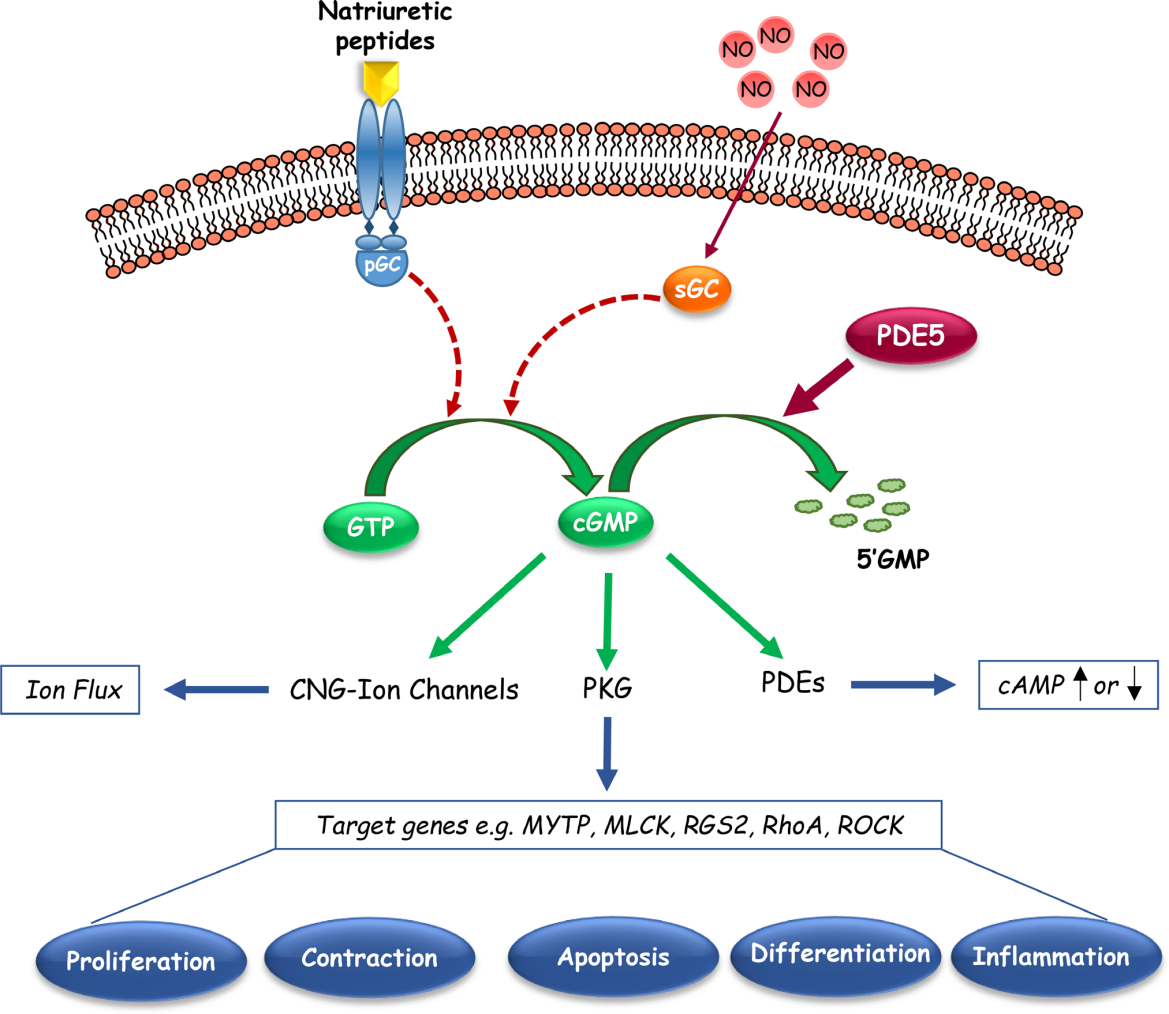
Schematic representation of cyclic guanosine monophosphate (cGMP) signaling pathways This graphic shows the basic synthetic, regulatory and downstream signalings that mediate the effects of endogenous cGMP in cells. Cyclic nucleotide phosphodiesterase type 5 (PDE5), which catalyzes the hydrolytic breakdown of cGMP into its biologically inactive derivative, regulates the amplitude and the duration of cGMP signalling. pGC, particulate Guanylyl Cyclase; sGC, soluble Guanylyl Cyclase; CNG-Ion channels, cyclic nucleotide-gated Ion channels; PKG, cGMP-dependent Protein Kinase or Protein Kinase G; MYTP, Myosin Phosphatase Targeting Subunit; MLCK, Myosin Light Chain Kinase; RGS2, Regulatory of G-coupled Signaling 2; ROCK, Rho-kinase.

In catalysing the hydrolysis of cGMP, PDE5 plays critical roles in controlling its intracellular levels, the compartmentalization of its signalling pathways and its downstream biological responses. cGMP signalling is schematically shown in Figure 2. Briefly, cGMP activates different pathways, resulting into the activation of cGMP-dependent protein kinase G (PKG), cyclic nucleotide-gated (CNG) ion channels, or certain cGMP-binding PDEs, which lead to protein phosphorylation, ion fluxes, or cyclic nucleotide hydrolysis to affect gene expression or other aspects of cellular activity [99]. An essential player in cGMP signaling is considered the serine/threonine protein kinase PKG, whose downstream substrates are implicated in a variety of biological processes, including calcium homeostasis, platelet activation and adhesion, smooth muscle contraction, cardiac function, vasodilation, cell differentiation, proliferation, and apoptosis [100, 101]. The most recognized function of PDE5A is the modulation of vascular tone via regulation of intracellular cGMP and calcium levels, particularly in the lung and penis [75]. Moreover, cGMP-PKG signalling pathway activation increases cell proliferation and permeability in the vascular endothelium [102–104]; whereas it negatively impacts hypertrophy and contractility in cardiac myocardium [105, 106]. PDE5 has been also involved in the regulation of platelet aggregation [107], and in the improvement of learning and memory processes [108].

### PDE5 inhibitors: selectivity and adverse effects

Since the discovery and characterization of PDE5, a collection of molecules able to inhibit its enzymatic activity have been conceived. The first drugs, such as IBMX (3-isobutyl-1-methylxanthine), dipyridamole and coffee, have been shown to be potent but non selective inhibitors. Later, zaprinast was described as a more specific molecule [109], but it did not find its application into clinical practice. However, its chemical structure was used to design compounds with higher potency and selectivity, leading to the identification in 1989 of sildenafil (1-[4-ethoxy-3-(6, 7-dihydro-1-methyl–7-oxo-3-propyl-1H-pyrazolo [4, 3-d]pyrimidin-5-yl) phenylsulphonyl]-4-methylpiperazine). Sildenafil, developed and marketed as Viagra by Pfizer, showed a higher selectivity (> 1000 fold) for human PDE5 over PDE2, PDE3 and PDE4 and moderate selectivity (> 80 fold) over PDE1 [110]. On the contrary, this drug was shown to be only ~ 10 fold more effective for PDE5 than retinal PDE6, explaining some blue tinting of vision reported as adverse effects after its use [111]. As a consequence, many companies designed other selective PDE5 inhibitors with reduced inhibitory effects towards PDE6, including vardenafil (Levitra, Bayer-GSK), and tadalafil (Cialis, IC351, Lilly-ICOS), which is 1000 fold less potent towards PDE6 [112]. These compounds also interact with PDE11, with tadalafil being the most potent of them in respect to vardenafil (IC50 = 73 nM and IC50 = 840 nM, respectively), but the functional consequences of these effects are still unknown [113]. Compared to sildenafil and vardenafil, tadalafil also provides a longer therapeutic effect due to its extended half-life [114]. A low incidence of some PDE-related adverse events was then obtained with avanafil (Stendra, Vivus inc.) [115]. Indeed, this molecule, which acts more rapidly than other PDE5 inhibitors, showed a higher selectivity against non-PDE5 isozymes as following: i) ~ 6.5 fold greater than sildenafil and vardenafil for PDE6; ii) more than 27 fold compared to sildenafil for PDE1; iii) ~ 760 fold higher than tadalafil for PDE11. Other PDE5 inhibitors, including udenafil, mirodenafil and lodenafil carbonate are currently under clinical investigation [116–118]. It is important to underline that some PDE5 inhibitors may also affect non-PDE proteins. Indeed, it has been shown that vardenafil was able to block calcium channels in rabbit pulmonary arteries and human platelets [119], and sildenafil interacted with multidrug resistance protein 1 (MDR1; also known as ABCB1) and antigen peptide transporter 1 (APT1; also known as ABCB2) to block drug extrusion from cells [120]. This latter off-target effect could be significant in reducing ABCB1- and ABCB2-mediated drug resistance and thus improving the efficacy of some chemotherapeutic agents, most probably independently of PDE5 inhibition [120, 121].

Data from reports using selective PDE5 inhibitors have been essential for a robust understanding of the cellular functions that are regulated by PDE5 in physiological states as well as under pathological conditions. For instance, sildenafil, originally designed as an antihypertensive molecule or a coronary vasodilator, was shown to induce responses in off-target tissues, such as in penis, changing the focus of this agent to erectile dysfunction (ED) and proposing PDE5 as a target for the treatment of ED. In addition, the improvement of pulmonary vascular physiology observed in *in vitro* and *in vivo* models of pulmonary hypertension following PDE5 inhibition has provided the rationale to recommend also PDE5 as a target for the treatment of pulmonary hypertension and respiratory distress [122, 123].

At the present time, the orally administrated PDE5 inhibitors sildenafil and tadalafil have Food and Drug Administration approval for the treatment of ED as well as pulmonary arterial hypertension (PAH); whereas vardenafil and avanafil are approved only for ED. However, a great number of papers published over the last decade highlighted the potential clinical use of PDE5 inhibitors in other applications, for which current therapies are limited and in which the mechanism of action does not necessarily rely on their known vasodilatatory effects [124]. Table 2 summarizes the medical conditions other than ED and PAH that have shown consistent benefits from treatment with these pharmacologic agents [124–196]. Importantly, reported adverse effects are generally mild, i.e. flushing, headache, backache, dyspepsia, and nasal congestion. Some users of sildenafil and vardenafil, that, as mentioned earlier, slightly inhibit photoreceptor PDE6, may also experience temporary and reversible minimal visual disturbances. Therefore, as reported in various reviews of pharmacotherapy, they represent a class of relative benign molecules in terms of safety and tolerability [197–199].

**Table 2:** Proposed novel applications of PDE5 inhibitors

Applications	Conditions	Agents	References
Male genitourinary dysfunctions	Benign prostatic hyperplasia and lower urinary tract symptoms Peyronie's disease Priapism Premature ejaculation, inability to ejaculate Low sperm count or motility	Sildenafil, Tadalafil, Vardenafil, UK-369, 003 Sildenafil Sildenafil Tadalafil, Sildenafil, Vardenafil Tadalafil, Sildenafil, Vardenafil	[125–130] [131–134] [135–137] [138–140] [141–145]
Neurologic dysfunctions	Neurogenesis and recovery from stroke Cognitive functions	Tadalafil, Sildenafil Sildenafil	[146–149] [150–153]
Tissue and organ protection	Antineoplastic agent toxicity Gastrointestinal damage	Sildenafil Sildenafil	[157–157] [158–160]
Cutaneous Ulcerations	Antiphospholipid syndrome Scleroderma Refractory Raynaud's phenomenon Systemic sclerosis	Sildenafil Sildenafil Sildenafil Sildenafil	[161] [162, 163] [163–165] [166, 167]
Transplant and reconstructive surgery	Heart transplant Liver transplant Renal transplant Lung transplant Reconstructive surgery	Sildenafil Sildenafil Sildenafil Sildenafil Sildenafil	[168–170] [171–174] [175] [176] [177–178]
Female genital dysfunctions	Fertility Pre-eclampsia	Sildenafil Sildenafil	[179–184] [185–187]
Diabetes	Neuropathy and vasculopathy Endothelium damage	Sildenafil Sildenafil, Tadalafil	[188–191] [192–196]

### Links between PDE5 and selected cancers

**Figure 3:**
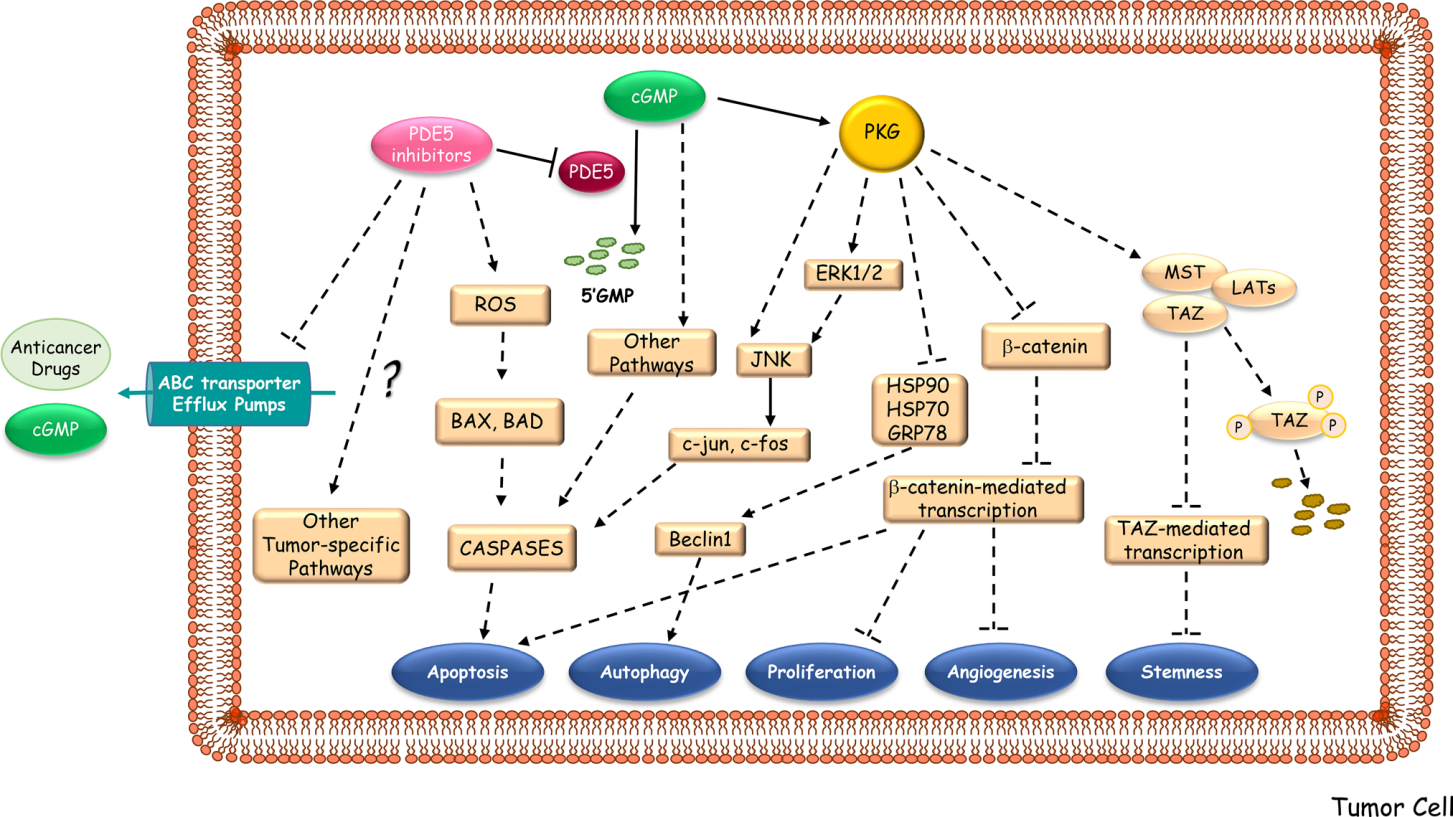
Proposed mechanisms underlying the anti-cancer activities of PDE5 inhibitors PDE5 inhibitors may hamper tumor progression by activating downstream signaling pathways, mainly PKG-mediated ones, which induce apoptosis, autophagy, growth suppression, inhibition of angiogenesis and of stemness. PDE5 inhibitors may also enhance the therapeutic effectiveness of multiple anti-neoplastic agents by increasing intracellular accumulation of drugs and cGMP levels through the block of the substrate efflux function of ABC multidrug-resistant transporters. ABC transporter Efflux Pumps, ATP-binding cassette transporter Efflux Pumps; ROS, Radical Oxygen Species; HSP, Heat Shock Protein; GRB78, Glucose-Regulated Protein; JNK, c-Jun N-terminal kinases; ERK, Extracellular Signal-regulated Kinases; MST, Mammalian Ste20-like Protein Kinase; LAT, Large Tumor Suppressor Kinase; TAZ, Transcription Regulator Protein-1.

There has been a remarkable interest in identifying new clinical use of PDE5 inhibitors as potent anticancer drugs with a novel mechanism of action (Figure 3). Since 2000, more than 150 papers have been reported on the connection of PDE5 and different kind of tumors. Indeed, increased expression of PDE5 in various human malignancies and the lack of such expression in normal cells have been described. In addition, PDE5 inhibitors have been examined for their direct inhibitory effects on tumor cell lines, for their ability to act as a sensitizers of cancer cells to chemotherapeutic agents, and as cancer chemopreventive agents [12]. However, much of the research is preclinical, and only few clinical trials have been completed or are ongoing so far (http://www.clinicaltrials.gov). The list of the studies focused on the effects of PDE5 inhibition in cancer patients is reported in Table 3.

**Table 3:** List of the studies on PDE5 inhibitors and cancer at www.clinicaltrials.gov^*^

Inhibitor	Indication	Status	ClinicalTrials.gov^#^
Sildenafil	Pancreatic Cancer, Cholangiocarcinoma Advanced Solid Tumors Bladder Cancer Kidney Cancer Breast, Gastrointestinal, Genitourinary, Gynecological Cancers Non-small Cell Lung Cancer High-grade Glioma Waldenstrom's Macroglobulinemia	Phase 1 (recruiting) Phase 1 (recruiting) Phase 2 (recruiting) Phase 2 (completed) Phase 1 (active, not recruiting) Phase 2, Phase 3 (completed) Phase 2 (recruiting) Phase 2 (completed)	NCT02106871 NCT02466802 NCT02422277 NCT01950923 NCT01375699 NCT00752115 NCT01817751 NCT00165295
Tadalafil	Head and Neck Squamous Cell Carcinoma Head and Neck Cancer Head and Neck Cancer Head and Neck Squamous Cell Carcinoma Multiple Myeloma Multiple Myeloma Prostate Cancer Pancreatic Cancer Pancreatic Cancer Primary Abdominal Malignancy	Phase 1 (recruiting) Phase 2 (recruiting) Phase 3 (completed) Phase 2 (completed) Phase 2 (recruiting) Phase 2 (terminated) Phase 3 (completed) Phase 1 (active, not recruiting) Phase 1 (active, not recruiting) Phase 1 (not yet recruiting)	NCT02544880 NCT02544880 NCT00843635 NCT00894413 NCT01858558 NCT01374217 NCT00931528 NCT01342224 NCT01903083 NCT02998736
Vardenafil	Glioma, Brain Neoplasms, Brain Metastasis	Early Phase 1 (recruiting)	NCT02279992
Udenafil	Sigmoid Colon and Rectal Cancers	Phase 2 (completed)	NCT00607282

### Lung cancer

Lung cancer has emerged as the most common cancer in the world, both in terms of new cases and deaths (1.82 million diagnoses and 1.6 million deaths recorded in 2012) with the highest rates in Central/Eastern Europe and Eastern Asia [2]. Non-small cell lung cancers (NSCLCs) represent more than 80% of all lung cancers [200]. Despite current advances in chemotherapy treatment regimens, response rates are still < 50%, and complete remissions remain rare, highlighting the need of agents with novel mechanisms of action to improve clinical outcomes.

Over the last years, the apoptotic and growth inhibition activities of PDE5 inhibitors have been demonstrated in numerous lung cancer cell lines. Most of the experiments were performed by using exisulind (sulindac sulfone), a sulfone metabolite of the nonsteroidal anti-inflammatory drug (NSAID) sulindac. Exisulind, which lacks the hallmark cyclooxygenase inhibitory activities of NSAIDs, acts as a cGMP PDE inhibitors, causing a persistent increase in cellular cGMP, and inducing PKG [201–203]. It was previously demonstrated that the combination of exisulind with cytotoxic drugs resulted in a synergistic inhibition of human lung cancer cell growth in culture [204]. In orthotopic lung cancer model systems, exisulind in association with docetaxel significantly induced apoptosis, reduced tumor growth and metastasis, and increased survival [10, 205, 206]. PDE5 inhibitors also improved the chemosensitivity of anti-cancer agents by increasing endocytosis-mediated cellular drug uptake in lung cancer cells; for instance, oral administration of the PDE5 inhibitor vardenafil significantly increases the accumulation and enhances the anti-tumor effect of trastuzumab in a xenograft mouse model of lung cancer [207]. Another study showed that sildenafil was able to enhance the anti-tumor effects of the standard of care drug pemetrexed in NSCLCs. In two models of human NSCLC cells growing in athymic mice, it was found that pemetrexed and sildenafil interacted in an additive fashion to suppress tumor growth and this effect was further enhanced *in vivo* by co-treatment with the mTOR inhibitor temsirolimus [208]. The complex molecular mechanisms by which these drug combinations induce lung cancer cell death were via increasing toxic autophagosome formation, and through the activation of extant death receptors. More recently, Booth et al. have evidenced how sildenafil enhanced the lethality of pemetrexed and sorafenib, a potent inhibitor of chaperone ATPase activities, in multiple genetically diverse lung cancer cell lines and in xenografts of lung cancer in athymic nude mice [208].

However, the clinical evidences do not support experimental findings. In 2006, a phase I/II study was designed to evaluate the safety and efficacy of exisulind in combination with gemcitabine as second-line therapy in NSCLC patients whose disease progressed more than 3 months from completion of first-line chemotherapy [209]. Although the primary endpoint of improving time to progression was met, the overall survival outcome of patients treated with the two drugs appears only slightly better than other phase II studies of single-agent gemcitabine. This may be likely due to suboptimal dosing of exisulind, or suboptimal scheduling or the lack of functional interaction between exisulind and cytotoxic drugs despite the preclinical observations. Other phase II studies using exisulind in combination with chemotherapy in advanced NSCLCs show no objective benefits regarding both overall survival and time to progression compared with historic controls treated with chemotherapy alone [210–212]. Disappointing results were also reported for the combination of carboplatin, etoposide, and exisulind as initial therapy for patients with newly diagnosed extensive stage small cell lung cancers [213].

### Prostate cancer

Prostate cancer represents the second most frequently diagnosed solid malignancy among men worldwide, accounting for about 1.1 million cases in 2012 [1]. Fortunately, due to early detection and the nerve-sparing prostatectomy, relative death rates have been decreasing and men have been given hope for recovery of bladder and erectile function. Penile rehabilitation after prostatectomy often includes treatment with PDE5 inhibitors and accordingly, association between the effects of these drugs and cancer recurrence have been examined in different experimental and clinical studies.

In support of PDE5 inhibitors’ ability to affect prostate carcinogensis, it was reported that exisulind suppressed the growth of metastatic, human prostate cancer cells in a nude mouse xenograft model by increasing apoptosis [214]. In contrast, Qian et al. noted that incontinuous oral administration of sildenafil citrate was not able to influence primary tumor growth and metastasis in an orthotopic prostate cancer model, most probably because cGMP elevation were only transient [215]. It was shown that sildenafil enhanced apoptosis and antitumor efficacy of doxorubicin in mice bearing prostate tumor xenografts, while attenuating its cardiotoxic effects (i.e. left ventricular dysfunction) [216]. Next, the same research group investigated the mechanism by which sildenafil may sensitize prostate cancer cell to doxorubicin-mediated apoptosis, showing CD95 (death receptor Fas)/FLIP (Fas-associated death domain FADD) as key regulators of this event [217]. In 2016, by *in vitro* cell culture and *in vivo* xenograft approaches, it was clearly demonstrated that PDE5/cGMP/PKG signal targets to Hippo/TAZ pathway in maintaining stemness of prostate cancer stem cells, evidencing a novel role of PDE5 in governing stem cell features [218]. Moreover, PDE5 was found to be mainly located in the stromal compartment of the prostate, and accordingly tadalafil reduced proliferation of primary prostate stromal cells at a greater extent than of primary prostate basal epithelial cells [219]. Tadalafil also attenuated TGFβ1-induced fibroblast-to-myofibroblast trans-differentiation, suggesting a potential value of PDE5 inhibitors in preventing stromal enlargement and treating benign prostatic hyperplasia ([219] and reviewed in [220]).

A retrospective analysis including a total of 4974 men revealed that the use of PDE5 inhibitors over a 7-year period was associated to a decreased incidence rate of prostate cancer among men with ED compared to men with ED of the same age and with similar risk factors who were not treated with these agents [221]. It was hypothesized that a higher ejaculation frequency can protect against prostate cancer development or alternatively vasodilation induced by PDE5 inhibition may counteract hypoxia and thereby thwart the emergence of more aggressive cancer phenotypes [222–224]. Another group also evaluated the safety and efficacy of exisulind (twice daily for 12 months) in delaying disease progression in men with rising prostate specific antigen (PSA) levels after radical prostatectomy and found that exisulind inhibited the increase in PSA overall and prolonged PSA doubling time in high-risk patients compared with placebo [225]. Contrary to these findings, using a large clinical database of patients with prostate cancer (*n* = 4752) with a median follow-up of 60.3 months, it was shown that the use of PDE5 inhibitors after radical prostatectomy may adversely impact biochemical recurrence (BCR), defined as a PSA of 0.2 ng/ml or greater and increasing after radical prostatectomy [226]. However, there are several limitations to this study, such as the lack of information on the type of the drug, the dose, the exact duration and frequency of use. Other authors retrospectively performed a similar analysis of BCR in 2579 patients treated with bilateral nervesparing radical prostatectomy and showed that there was no association between PDE5 inhibitor use and an increased risk of BCR, regardless of the therapeutic regimen used [227].

On the basis of these findings, there is no strong evidence to modify current clinical practice, and therapy with PDE5 inhibitors still remains the more recommended option for many postprostatectomy patients for penile rehabilitation programs [228].

### Breast cancer

Breast carcinoma is the most common malignancy and the leading cause of cancer-related death in women worldwide. Breast cancer is a complex and highly heterogeneous disease classified on the basis of global gene expression analyses into at least five biologically different intrinsic subtypes (i.e. luminal A, luminal B, human epidermal growth factor receptor 2 (HER2)-enriched, basal-like, and normal-like) with distinct morphologic features, variable clinical outcomes and disparate therapeutic responses [229].

Increased PDE5 expression has also been reported in various cell lines deriving from breast cancer (MCF-7, HTB-26, MDA-MB-468) [54], giving the rational to assess the anticancer effects of PDE5 inhibition. Indeed, the PDE5 inhibitor exisulind selectively exerted, in various breast cancer cells, pro-apoptotic and anti-proliferative effects concomitantly with elevation of cGMP and activation of PKG, without effects on human mammary normal epithelial cells [230]. Inhibition of PDE5 and activation of PKG by exisulind was associated with reduced oncogenic Wnt/β-catenin-mediated transcriptional activity and subsequent downregulation of target genes, including cyclin D1 and survivin [231]. Stable PDE5 silencing in the aggressive human breast cancer cell line MDA-MB-231T lead to a reduction of cell motility *in vitro* and of lung metastasis formations in an experimental metastasis assay *in vivo* [232]. This well fits with our recent data showing that stable overexpression of PDE5 in MCF-7 breast cancer cells significantly increased motility and invasion of all the stable PDE5- transfected clones tested compared to parental cells [7]. In addition to the direct anti-cancer activities, it was demonstrated that PDE5 inhibitors may act as potential cancer chemopreventive agents, due to their ability to suppress 1-methyl-1-nitrosourea (MNU)-induced mammary carcinogenesis [233].

In breast cancer clinical samples, increased PDE5 expression was verified by both RT-PCR and immunohistochemistry analyses, and was correlated with tumor grade stage and lymph node involvement [9, 234]. More recently, we demonstrated that PDE5 was differentially expressed among breast cancer molecular subtypes, with higher levels in HER2-enriched and triple-negative subtypes compared to the more favourable estrogen receptor (ER)-positive Luminal B- and the Luminal A subtypes [7]. Importantly, high PDE5 expression predict a worse prognosis for a cohort of 1,988 patients at 8-year median follow-up [7]. Significant difference was also found between overall survival for ER-positive patients having high or low PDE5 levels, highlighting a role for PDE5 in predicting disease progression in ER-positive tumors that according to our immunohistochemistry analysis may have lower levels of the enzyme compared with ER-negative cases. In addition, since PDE5 retained its significance when performing a multivariate analysis including PDE5 expression, ER, HER2, and lymph node status in the entire database, it is tempting to speculate that high PDE5 levels may independently predict poor outcome among patients with breast cancer. However, up to now, only one study was conducted to clinically evaluate the safety and activity of PDE5 inhibitor in breast cancer. In particular, it was demonstrated that administration of exisulind in combination with capecitabine was well tolerated in a small number of patients with metastatic breast cancer (*n* = 35), but the synergism between these two drugs at the doses tested appears to be modest [9].

### Colorectal cancer

Colorectal cancer is the third most commonly diagnosed cancer in men and the second in women worldwide, with an estimated 1.4 million cases and 693,900 deaths occurring in 2012 [1]. In contrast to incidence trends, decreasing colorectal cancer mortality rates have been observed in a large number of countries and are most likely attributed to colorectal cancer screening, reduced prevalence of risk factors, and/or improved therapies. However, when widespread malignancy is encountered, these cases are not responsive to curative treatments.

In several colon tumor cell lines (e.g. HT29, T84, and HCT116), exisulind and analogs/derivatives were able to inhibit the oncogenic activity of β-catenin through a direct suppression of its transcription or increased proteosomal degradation, thereby promoting cell death [202, 235–238]. It was also reported that sulindac metabolites inhibit the mitogen-activated protein/extracellular signal-regulated kinase kinase (MEK/ERK) signaling cascade in colorectal cancer cell lines at doses that induce apoptosis, as additional molecular mechanisms by which Sulindac inhibits tumor cell growth [239, 240]. In addition, treatment of human colorectal cancer cells with sildenafil resulted in cell proliferation inhibition, cell cycle arrest and apoptosis accompanied by increased intracellular reactive oxidative specie levels *in vitro* and caused the reduction of xenograft tumor growth in nude mice [241]. Recently, combined inhibition of PDE5 and PDE10 by treatment with PDE isozyme-selective inhibitors, or by siRNA knockdown was shown to suppresses β-catenin, and the levels of its downstream targets, thereby suppressing proliferation and inducing apoptosis in colon tumor cells [242].

In clinical studies, exisulind prevented colorectal polyp formation in patients with familial adenomatous polyposis (FAP) over 24 months [243, 244]. Moreover, it has been demonstrated that exisulind inhibited azoxymethane-induced colon carcinogenesis in rats [201] and sildenafil suppressed polyp formation and inflammation in mice treated with azoxymethane/dextrane sulfate sodium [245], highlighting the chemopreventive role of PDE5 inhibition [246].

### Brain cancer

Cancers of the brain and central nervous system accounted for 256,000 new cases and 189,000 deaths in 2012, with the highest incidence and mortality rates in more developed regions [2].

In both neuroblastoma N18TG2 and hybrid neuroblastoma-glioma NG108–15 cells, the presence and regulation of PDE5 mRNA during cell differentiation was previously demonstrated [247]. In medulloblastoma cells, PDE5 inhibitors interacted in a greater than additive fashion with vincristine/etoposide/cisplatin to cause cell death. PDE5 inhibitors promoted autophagy and enhanced chemotherapy-induced DNA damage in a nitric oxide synthase-dependent fashion [248]. Pharmacologic modulation of cGMP signaling is emerging as a novel approach for enhancing therapeutic agent permeability across the blood-brain tumor barrier, thereby increasing delivery to brain tumors and metastases. Oral administration of sildenafil and vardenafil selectively improved tumor capillary permeability in 9L gliosarcoma-bearing rats, without changes in normal brain capillaries [249]. Importantly, tumor-bearing rats treated with the chemotherapy agent, adriamycin, in combination with vardenafil exhibited a survival significantly longer than rats treated with adriamycin alone [249]. The combination of OSU-03012/sildenafil synergized with low concentrations of sorafenib to kill glioblastoma cells *in vitro* and *in vivo* [250]. PDE5 inhibitors enhanced transport and therapeutic efficacy of trastuzumab in hard-to-treat brain metastases from different primary tumors [251]. Tadalafil can also enhance the treatment efficacy of the chimeric anti-CD20 monoclonal antibody Rituximab by improving the microvascular permeability in intracranial brain lymphoma mice model [252]. In contrast to these findings, it was shown that genetic and pharmacological inhibition of PDE5 activity strongly enhanced cell motility and invasiveness in human glioblastoma T98G cells, whereas PDE5 overexpression in PDE5-negative U87G cells significantly inhibited their invasive potential and interfered with DNA damage repair and cell survival following irradiation [253].

Analysis of a cohort of 69 patients affected by glioblastoma multiforme (GBM) who underwent chemotherapy and radiotherapy following surgical resection of the tumor revealed that PDE5 was strongly expressed in 50% of cancer cases [253]. Retrospective analysis indicated that high PDE5 expression significantly correlated with increased overall survival of patients, identifying this enzyme as a favourable prognostic marker for GBM, which negatively affects cell invasiveness and survival [253].

### Thyroid cancer

Carcinoma of the thyroid is the most common malignancy of endocrine organs, representing 2.1% of the new cancer cases (about 230,000 among women and 70,000 among men in 2012) [254]. Over the last few decades, a steady increase of thyroid cancer has been appreciably observed in most areas of the world, and if current trends are maintained, thyroid cancer may become the fourth most common cancer by 2030. More than 95% are well-differentiated papillary or follicular tumors that derive from follicular epithelial cells and can be effectively managed by surgical resection with or without radioctive iodine ablation. A minority of thyroid cancers are medullary thyroid carcinomas that derive from the neuroendocrine C cells of the thyroid or anaplastic malignancies that are the rarest but the most lethal subtype [255]. Treatment for about two thirds of patients with progressive metastatic papillary and follicular thyroid cancers as well as for patients with less differentiated tumors is often of limited benefit, due to their inability to concentrate radioiodine or to the development of therapy resistance [256, 257]. Thus, there is a pressing need for innovative treatments in patients having high risk of disease-related death.

The role of cGMP levels and PDE5 in thyroid cancer are still not well defined. Analysis of the mRNA expression of members of the 11 known families of PDEs has revealed the expression of PDE4, PDE5, PDE7 and PDE8 subtypes in normal thyroid tissues [258]. Previous studies have also reported the presence of PDE4 in toxic adenomas characterized by mutations in the TSH receptor (TSH-R) gene [259]. In 2015, Sponziello et al. showed, for the first time, higher mRNA and protein expression levels of PDE5 in a series of human papillary thyroid carcinomas belonging to two independent cohorts compared to non-tumor tissues [260]. Interestingly, tumors presenting BRAF V600E mutation, that is the most frequent genetic alteration and also a marker of aggressiveness [261], exhibited a marked upregulation of PDE5 respect to those without mutation. Increased PDE5 transcripts were also associated with a reduction of the expression of TSH-R, thyroglobulin (Tg), thyroid peroxidase (TPO), sodium/iodide symporter (NIS), important differentiation markers implicated in intra-thyroidal iodine metabolism and thyroid hormone synthesis. More recently, a second study reported higher intracellular cGMP levels and cGMP-PDE activity in thyroid malignant (papillar and follicular) carcinomas than in controls and benign pathologies (i.e. benign struma, adenomatous hyperplasia, chronic thyroiditis, benign adenoma) [262]. However, the cGMP-PDE expression was elevated in papillary carcinomas without lymph node involvement (N-) in respect to those with lymph node invasion (N±), while cGMP levels displayed an inverse trend. These events may represent an autophagic defence mechanism of the body against the cancer that is expanding and invading other tissues and organs. Although these findings are promising, further studies are needed to draw definitive conclusions.

In papillary and anaplastic thyroid cancer cells *in vitro*, sildenafil and tadalafil determined a reduction of proliferation and at lower doses, they were also able to reduce cellular migration [260]. The effects of PDE5 inhibitors were stronger in the cancer cell lines carrying the BRAF mutation, suggesting that these tumors could be a preferential subtype for the action of PDE5-targeted drugs. A significant decrease of the drug dosages necessary to achieve an anticancer effect was obtained with the encapsulation of these compounds within nanoliposomes [263], that may represent, if confirmed *in vivo*, a valuable novel formulation for the treatment of thyroid carcinomas and other types of cancers.

### Melanoma

Cutaneous melanoma accounted for almost 5% of all new diagnosed cancer cases, with a reported mortality of approximately 2%, making it the deadliest form of skin cancer. Although early detection carries an excellent prognosis, with surgical excision often being curative, the long-term survival rate for patients with metastatic melanoma is only 5% [264]. The disease derived from genetically altered epidermal melanocytes that arises because of complex interactions between genetic alterations, such as RAS pathway mutations and environmental factors, especially exposure to UV radiation [265]. The role of PDE5 on pathogenesis or progression of melanoma remains still an area of debate. Murata et al. have shown PDE5 activity and expression in malignant melanoma cells, and reported an important function for this enzyme in regulating melanoma progression as two PDE5 inhibitors inhibited cell growth [266]. However, the first mechanistic insights on the link between PDE5 and melanoma was suggested by Arozarena et al. in 2011 [267]. Indeed, it was demonstrated that in melanoma cells oncogenic BRAF, through the transcription factor BRN2, was able to suppress PDE5A expression. PDE5 down-regulation, in turn, leads to enhanced cGMP levels, which induce an increase in cytosolic Ca^2+^, a stimulation of actin-myosin contractility and a subsequent increase in cell invasion *in vitro* and *in vivo* [267]. Accordingly, immunohistochemistry analysis in a tissue microarray consisting of triplicate cores of 28 primary and 29 metastatic malignant melanoma cases revealed a statistically significant downregulation of PDE5A in metastatic tumors [267]. More recently, it was reported that the growth-promoting cGMP signaling could be potentiated pharmacologically by treatment with sildenafil in murine and human melanoma cells, suggesting that possible skin adverse effects of PDE5 inhibitors should be better considered [268].

Clinically, in a prospective cohort study, men who used sildenafil for ED had a statistically significant elevated risk of developing melanoma, without affecting the risk of squamous or basal cell carcinomas, and this correlation was maintained in the models controlling for the major host characteristics, such as age, body mass index, family history, sun exposure, and UV index in the state of residence [269]. A subsequent nationwide, population-based, nested case-control study in Sweden, including 4065 melanoma cases diagnosed from 2006 through 2012, showed that the use of PDE5 inhibitors was associated with a modest but statistically significant increased risk of malignant melanoma, but the pattern of association (e.g. the lack of association with multiple filled prescriptions) raises questions about the causality of this relationship [270]. Later, a large UK-based primary care database, with 145,104 men who were prescribed a PDE5 inhibitor and 560,933 matched controls, was examined, but the findings showed no evidence of a positive association between PDE5 inhibitor exposure and melanoma risk after matching or adjusting for key potential confounders [271]. More recently, a meta-analysis of 5-population-based observational studies revealed an increased risk of malignant melanoma in users of PDE5 inhibitors for ED [272]. Although a large number of patients and a long follow-up were included in this report, population selective bias, the lack of dose-response analysis and in general the weakness inherent in observational studies should be acknowledged. Thus, collectively, the present findings are insufficient to alter current clinical recommendation.

### Other cancers

A possible role of PDE5 inhibition has been suggested also in the management of other cancers. Immunohistochemistry showed that PDE5 was overexpressed in human squamous and transitional cell carcinomas compared with normal urothelium and accordingly, exisulind exhibited antineoplastic activity *in vivo* in a model of rat urinary bladder tumorigenesis [8]. The addition of PDE5 inhibitors to multiple existing treatment regimens, including doxorubicin, mitomycin C, gemcitabine, cisplatin and paclitaxel, significantly enhanced chemotherapy lethality by stimulating the extrinsic apoptosis pathway via CD95 and by promoting autophagy through RIP-1 (receptor interacting protein 1) in bladder and pancreatic cancer cell lines [156]. In human renal carcinoma cell lines, suppression of PDE5 gene expression by PDE5 siRNA reduced cell proliferation and induced apoptosis through cGMP-PKG activation [273]. Sildenafil in combination with C-type natriuretic peptide synergistically inhibited proliferation of rhabdomyosarcoma cells and suppressed tumor growth *in vivo* [274]. Sildenafil and vardenafil were also able to induce apoptosis in peripheral blood mononuclear cells isolated from fourteen patients with chronic lymphocytic leukemia through a caspase 3-dependent pathway [275]. Recently, it was shown that vardenafil potentiates the killing effect of the green tea polyphenol (–) epigallocatechin-O-3-gallate on leukaemia and multiple myeloma cells, without affecting normal cells [276, 277]. Interestingly, in a patient with end-stage relapsed/refractory multiple myeloma, the addition of tadalafil reduced myeloid-derived suppressor cell function, that was associated to anti-myeloma immune responses and clinical benefit [278]. In 2015, a randomized, prospective, double blinded, placebo controlled, phase II clinical trial to determine the activity of PDE5 inhibitors on immune function in head and neck squamous cell carcinoma (HNSCC) patients was conducted [279]. Results showed that tadalafil can reverse tumor-specific immune suppression in these patients, with important therapeutic potential.

### Concluding remarks

Over the last decades, researchers have elucidated the roles that impairment of cGMP signaling pathway by PDE5 activity inhibition plays in the regulation of tumor development, and progression. As a consequence, our knowledge on the link between PDE5 inhibitors and cancer biology has expanded, holding great promise for future use of these agents in several cancers. Although evident clinical controversial data come from patients affected by melanoma and glioblastoma multiforme, studies discussed in this literature review show that PDE5 inhibition could be associated with a decreased risk of cancer development and suppression of tumor progression in several malignancies including those of the lung, prostate, breast and colorectum. PDE5 inhibitors may also provide an additional antitumor immune response in patients affected by myeloma and head and neck squamous cell carcinomas. In addition, a synergistic effect with current chemotherapeutic regimens and monoclonal antibodies has been reported. However, this research suffered from the weakness of the clinical studies conducted until now that make difficult to draw a general conclusion. First, it would be important to distinguish class effects of PDE5 inhibitors versus effects unique to selective agents within the class, also in relation to non-specific inhibition of other PDEs as well as to potential off-target effects that can both positively and negatively influence the risk-benefit profile of PDE5 inhibitors. Secondly, we will need information concerning the optimal dosages of these molecules in the various applications, their monitoring, and the potential interactions with other agents regularly used in each patient population. Moreover, major clinical benefits will come from more definitive specifications of individual patient variables that may point toward the use of PDE5 inhibitors in general and each specific drug of this class, providing antitumor therapy with reduced adverse effects. Certainly, the clarification of additional molecular processes of potential oncogenic function of PDE5 as well as further clinical trials including PDE5 inhibitors will help to facilitate better applications of PDE5 targeting drugs in the area of cancer treatment.
